# Scope and Predictive Genetic/Phenotypic Signatures of Bicarbonate (NaHCO_3_) Responsiveness and β-Lactam Sensitization in Methicillin-Resistant Staphylococcus aureus

**DOI:** 10.1128/AAC.02445-19

**Published:** 2020-04-21

**Authors:** Selvi C. Ersoy, Mariam Otmishi, Vanessa T. Milan, Liang Li, Youngju Pak, Jose Mediavilla, Liang Chen, Barry Kreiswirth, Henry F. Chambers, Richard A. Proctor, Yan Q. Xiong, Vance G. Fowler, Arnold S. Bayer

**Affiliations:** a The Lundquist Institute, Torrance, California, USA; b California State University Dominguez Hills, Carson, California, USA; c Meridian Health, Nutley, New Jersey, USA; d UCSF School of Medicine, San Francisco, California, USA; e University of Wisconsin School of Medicine and Public Health, Madison, Wisconsin, USA; f Geffen School of Medicine at UCLA, Los Angeles, California, USA; g Duke University Medical Center, School of Medicine, Durham, North Carolina, USA

**Keywords:** antimicrobial susceptibility testing, beta-lactams, methicillin-resistant *Staphylococcus aureus*, sodium bicarbonate

## Abstract

Addition of sodium bicarbonate (NaHCO_3_) to standard antimicrobial susceptibility testing medium reveals certain methicillin-resistant Staphylococcus aureus (MRSA) strains to be highly susceptible to β-lactams. We investigated the prevalence of this phenotype (NaHCO_3_ responsiveness) to two β-lactams among 58 clinical MRSA bloodstream isolates. Of note, ∼75% and ∼36% of isolates displayed the NaHCO_3_ responsiveness phenotype to cefazolin (CFZ) and oxacillin (OXA), respectively. Neither intrinsic β-lactam MICs in standard Mueller-Hinton broth (MHB) nor population analysis profiles were predictive of this phenotype. Several genotypic markers (clonal complex 8 [CC8]; *agr* I and *spa* t008) were associated with NaHCO_3_ responsiveness for OXA.

## Abstract (precis)

Addition of sodium bicarbonate (NaHCO_3_) to standard antimicrobial susceptibility testing medium reveals certain methicillin-resistant Staphylococcus aureus (MRSA) strains to be highly susceptible to β-lactams. We investigated the prevalence of this phenotype (NaHCO_3_ responsiveness) to two β-lactams among 58 clinical MRSA bloodstream isolates. Of note, ∼75% and ∼36% of isolates displayed the NaHCO_3_ responsiveness phenotype to cefazolin (CFZ) and oxacillin (OXA), respectively.

## INTRODUCTION

Staphylococcus aureus is a serious community and nosocomial pathogen and a leading cause of bacteremia, infective endocarditis, and device-related infections (1, 2). Many of these infections are caused by methicillin-resistant S. aureus (MRSA), which is generally perceived to be “resistant” to those β-lactam antibiotic therapies used for the treatment of methicillin-susceptible S. aureus (MSSA) (3). MRSA exhibits *in vitro* resistance to oxacillin (OXA) by standard antimicrobial susceptibility testing (AST), which has been extrapolated to apply to all other β-lactams (excluding ceftaroline and ceftobiprole) (4). However, as shown recently, *in vitro* antimicrobial resistance of MRSA (and selected Gram-negative bacilli) may not always correlate to therapeutic resistance *in vivo* (5–8). Thus, efforts have been made to more effectively model the host environment in newer *in vitro* AST to improve the predictive power of these assays with regard to clinical outcomes (6, 7, 9). These modified AST protocols utilize tissue culture media or media that contain specific ions that a pathogen would consistently encounter within a host (e.g., sodium bicarbonate [NaHCO_3_]).

Recently, we identified a novel MRSA AST phenotype, termed “NaHCO_3_ responsiveness,” in which MRSA strains are highly susceptible *in vitro* to cefazolin (CFZ) and OXA in a medium supplemented with NaHCO_3_ (7). Prototype MRSA strains with this *in vitro* phenotype were also highly susceptible to these same β-lactams in an *ex vivo* simulated endocarditis vegetation model as well as in a rabbit model of infective endocarditis (7, 10). In contrast, *in vitro* NaHCO_3_-nonresponsive isolates were also resistant to CFZ and OXA under these same *ex vivo* and *in vivo* conditions.

The aim of this study was to delineate the frequency of the NaHCO_3_-responsive/nonresponsive phenotypes *in vitro* among a larger MRSA strain set. Thus, we analyzed a collection of 58 MRSA bloodstream isolates for the following to determine potential predictive markers of NaHCO_3_ responsiveness: (i) NaHCO_3_-responsive phenotypes to CFZ and OXA and (ii) linkage of this phenotype with other known phenotypic and genotypic markers. We identified a relatively large subset of NaHCO_3_-responsive strains to CFZ and/or OXA within this MRSA cohort.

(This study was presented in part at the IDWeek Conference, Washington, DC, October 2019 [11].)

## 

### Frequency of NaHCO_3_ responsiveness.

Among 58 clinical MRSA isolates, 22 were vancomycin- and daptomycin-susceptible bloodstream isolates obtained from the Cubist Isolate Collection. The remaining 36 strains (also vancomycin and daptomycin susceptible) were from bacteremic patients at Duke University Medical Center (kindly provided by Vance Fowler) (12). Fifty-five of the 58 isolates were β-lactamase positive as determined by nitrocefin disk assay as per manufacturer’s instructions (Becton, Dickinson).

**FIG 1:**
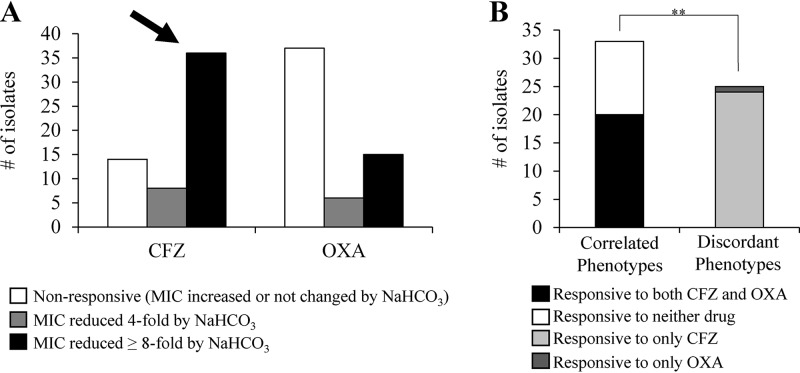
Frequency of NaHCO_3_ responsiveness among 58 clinical MRSA isolates. (A) Frequency of NaHCO_3_ responsiveness to cefazolin (CFZ) and oxacillin (OXA). Responsiveness is defined as a ≥4-fold reduction in MIC in the presence of NaHCO_3_ compared to that of medium lacking NaHCO_3_. (B) Frequency of coresponsiveness to CFZ and OXA. Coresponsiveness is defined as a strain that is NaHCO_3_ responsive to both CFZ and OXA. Correlated phenotypes are those in which a strain is either responsive to both drugs (coresponsive) or responsive to neither drug (nonresponsive). Discordant phenotypes are those in which a strain is responsive to only one drug (either CFZ or OXA). Kappa coefficient of correlation (κ) = 0.25, **, *P* = 0.008.

The NaHCO_3_-responsive phenotype to CFZ and OXA was defined as ≥4-fold reduction in MICs in the presence versus absence of 44 mM NaHCO_3_ by broth microdilution assay (7). Previously, we determined that this *in vitro* criterion was a good predictor of *in vivo* outcomes in a rabbit model of MRSA infective endocarditis (7). The majority of strains displayed NaHCO_3_ responsiveness to at least one of the two β-lactams (Fig. 1A; see also Table S1 in the supplemental material). Thus, 44/58 (76%) and 21/58 (36%) tested strains displayed reduced MICs to CFZ and OXA, respectively, in the presence of NaHCO_3_ (Fig. 1A). Additionally, the majority of such responsive strains displayed a ≥8-fold reduction in MICs in the presence of NaHCO_3_ (82% for CFZ and 71% for OXA) (Fig. 1A).

We identified 20/58 (35%) strains to be coresponsive to both CFZ and OXA, while 13/58 (22%) strains were responsive to neither drug (Fig. 1B). The significance of this correlation was calculated by the kappa coefficient (κ) for the coresponsive phenotype. Although the kappa coefficient indicated a positive correlation for this coresponsiveness, there were also a significant number of strains with discordant phenotypes (NaHCO_3_ responsive to only CFZ or only OXA) in our cohort sample (McNemar’s test; ****, *P* < 0.0001) (Fig. 1B). The majority of coresponsive phenotypes appeared to be driven by the 20/21 OXA-responsive strains that were also responsive to CFZ. These data indicate that testing for NaHCO_3_ responsiveness to OXA is a reasonable proxy for NaHCO_3_ responsiveness to CFZ but not *vice versa*.

Recent interest has focused on the use of the tissue culture medium, RPMI 1640, for AST as a more host-mimicking milieu, particularly for Gram-negative bacteria (13–15). In a previous study of five prototype MRSA strains, we determined that AST in RMPI 1640 (containing ∼25 mM NaHCO_3_) resulted in a ≥4-fold decrease in MICs to CFZ and OXA, despite only two of the five becoming susceptible to these same β-lactams in 44 mM NaHCO_3_-Mueller-Hinton broth (MHB) medium. Moreover, only the two NaHCO_3_-responsive strains were cleared *in vivo* from experimental endocarditis target tissues by these two β-lactams (7). To further assess the impact of RPMI medium on MRSA β-lactam MICs in the current cohort, 58/58 (100%) and 50/58 (86%) strains were rendered highly susceptible to CFZ and OXA, respectively, when tested in RMPI 1640 (see Table S1). Thus, RPMI medium does not appear to be a discriminative candidate as a host-mimicking medium for new MRSA AST.

### Population analysis profiles, time-kill synergy, and intrinsic (baseline) MICs of NaHCO_3_-responsive versus nonresponsive strains.

**FIG 2:**
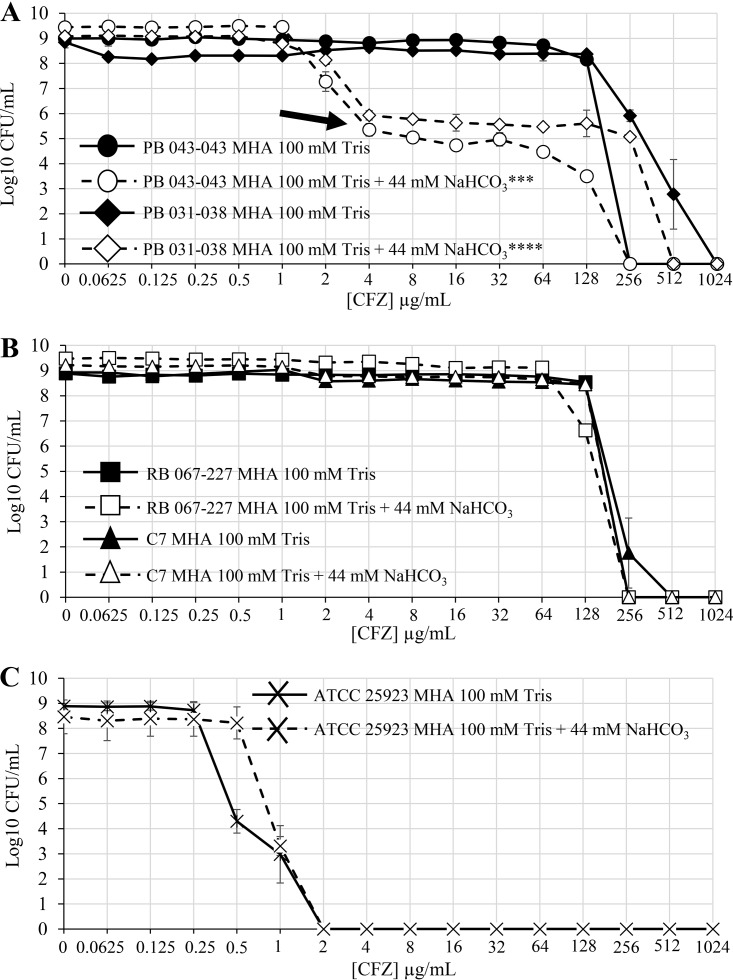
Population analysis profiles for NaHCO_3_-responsive and nonresponsive strains. (A) Population analysis profiles of CFZ for NaHCO_3_-responsive strains in the presence and absence of NaHCO_3_. The area under the curve (AUC), calculated by linear approximation, was significantly decreased by exposure to NaHCO_3_ in both strains as analyzed by Student’s *t* test (PB 043-043, ***, *P* < 0.001; PB 031-038, ****, *P* < 0.0001). (B) Population analysis profiles of CFZ for NaHCO_3_-nonresponsive strains in the presence and absence of NaHCO_3_. As analyzed by Student’s *t* test, there is no significant difference in the AUC for C7 in media ± NaHCO_3_; the AUC of RB 067-227 was significantly increased by exposure to NaHCO_3_ (*, *P* < 0.05). (C) Population analysis profiles of CFZ for the methicillin-susceptible S. aureus (MSSA) strain ATCC 25923.

Homoresistant MRSA strains are typically classified as those in which essentially the entire cell population displays the same level of resistance to an antibiotic of interest by population analysis profile (PAP) (16). In contrast, in heteroresistant strains, only a relatively small “subpopulation” of cells within the entire population display high-level resistance. Thus, PAPs were carried out on two responsive (PB 043-043 and PB 031-038) and two nonresponsive MRSA strains (C7 and RB 067-227) from our cohort in the presence and absence of NaHCO_3_ as previously described (7). All four strains exhibited a homoresistant CFZ PAP in standard Mueller-Hinton agar (MHA). Of note, both responsive strains demonstrated a significant repression of the overall resistant subpopulation in the presence of NaHCO_3_ (Fig. 2A). In contrast, neither nonresponsive strain PAP was impacted by growth in NaHCO_3_-containing medium (Fig. 2B). As expected, the PAP of an MSSA isolate determined in medium ± NaHCO_3_ was homogenously susceptible to CFZ in both conditions (Fig. 2C). Upon further PAP testing of the remaining strains within the cohort, a large proportion of responsive strains had homoresistant PAPs, with only two strains exhibiting heteroresistant PAPs (see Table S2 in the supplemental material). Similarly, the vast majority of nonresponsive strains tested had homoresistant PAPs, but this was not uniformly found among this group (Table S2). Thus, there was no obvious linkage between NaHCO_3_ responsiveness and heteroresistance (7) or NaHCO_3_ nonresponsiveness and homoresistance. These data underscore the notion that NaHCO_3_ responsiveness/nonresponsiveness is not merely a reflection of the proportion of the β-lactam-resistant subpopulations within a given MRSA strain; it is more likely that complex microbial factors are in-play.

**FIG 3:**
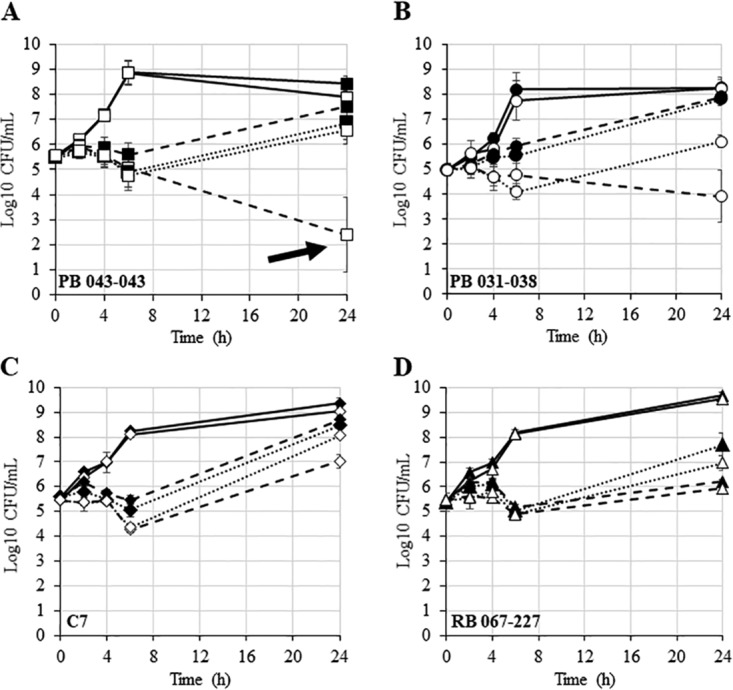
Time kill curves for NaHCO_3_-responsive and nonresponsive strains. (A) PB 043-043 (squares). (B) PB 031-038 (circles). (C) C7 (diamonds). (D) RB 067-227 (triangles). Closed symbols represent growth in CA-MHB 100 mM Tris, open symbols represent growth in CA-MHB 100 mM Tris + 44 mM NaHCO_3_, solid lines represent no drug control, dashed lines represent exposure to CFZ, and dotted lines represents exposure to OXA. Drug concentrations for PB 043-043 were 16 μg/ml for CFZ and 32 μg/ml for OXA; drug concentrations for all other strains were 64 μg/ml for both CFZ and OXA.

We assessed potential synergistic killing between NaHCO_3_ plus β-lactams in responsive versus nonresponsive strains by time-kill assay (7). Synergy was defined as a ≥2 log_10_ CFU/ml difference in counts at 24 h when comparing the β-lactam agent alone versus the combination of β-lactam plus NaHCO_3_. To qualify for synergy, there also had to be a bactericidal impact of the β-lactam plus NaHCO_3_ combination (i.e., ≥3 log_10_ CFU/ml decrease from the 0 h starting inoculum). In two responsive strains, only one strain (PB 043-043) exhibited both a bactericidal and synergistic impact of CFZ plus NaHCO_3_ (Fig. 3A and B). In contrast, neither nonresponsive strain displayed either a bactericidal or synergistic impact of CFZ or OXA plus NaHCO_3_ versus medium alone (Fig. 3C and D).

**FIG 4:**
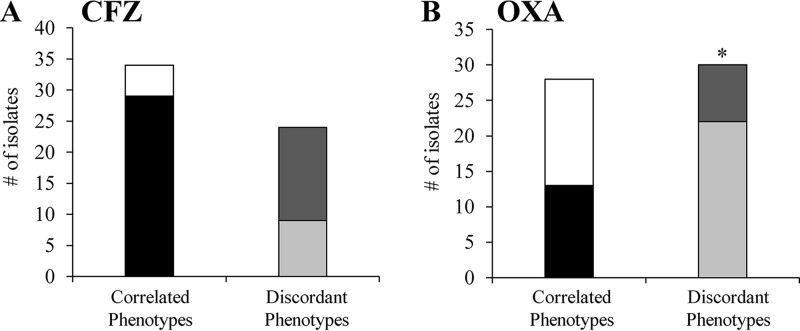
Relationship between intrinsic MIC and responsiveness for CFZ and OXA. (A) CFZ. (B) OXA. Both graphs show low intrinsic MIC/responsive (black), high intrinsic MIC/nonresponsive (white), low intrinsic MIC/nonresponsive (light gray), and high intrinsic MIC/responsive (dark gray). Relationship between intrinsic MIC and responsiveness for CFZ is random (κ = 0.01; 95% confidence interval (CI) = −0.24 to 0.27; Kappa coefficient and McNemar’s test, *P* > 0.05). Relationship between intrinsic MIC and responsiveness to OXA is also random with significant discordance (κ = 0.02; 95% CI = −0.21 to 0.25; Kappa coefficient, *P* > 0.05; McNemar’s test *, *P* = 0.01).

We assessed whether the NaHCO_3_-responsive phenotype might be predicted by a relatively low intrinsic (baseline) MIC to CFZ and OXA in standard MHB (i.e., an MIC in standard community-associated methicillin-resistant Staphylococcus aureus [CA-MHB] of <64 μg/ml). However, such lower intrinsic MICs were only randomly associated with NaHCO_3_ responsiveness for both drugs (Fig. 4A and B).

Taken together, the above data indicate that other intrinsic microbial properties, rather than the proportion of resistant cells within a population or the magnitude of that resistance, dictate the net bacteriostatic and/or bactericidal effects of CFZ and OXA in the presence of NaHCO_3_.

### Genotypic correlates of NaHCO_3_ responsivity.

We determined potential relationships between several well-characterized genotypic markers in MRSA with the NaHCO_3_-responsive phenotype for either β-lactam, employing clonal complex (CC) type, *agr* type, *spa* type, and staphylococcal cassette chromosome *mec* element (SCC*mec*) type. The CC, *agr*, *spa*, and SCC*mec* types were determined as previously described (17–20).

There was a broad range of CC types observed in our cohort (see Table S1); however, only CC types 5 and 8 provided a large enough sample size to make any statistical inferences. Neither of these two CC types was a statistically better predictor of responsiveness to CFZ (see Fig. S1A in the supplemental material). Interestingly, CC8 strains had a significantly higher proportion of OXA-NaHCO_3_-responsive strains than CC5 strains (Fig. S1B). Similar to what was observed in the CC type data, neither *agr* type I or II strains were a better predictor of responsiveness to CFZ (see Table S1; chi-square analysis; *P* = 0.9); in contrast, *agr* type I strains exhibited a significantly higher frequency of OXA NaHCO_3_ responsiveness than *agr* type II strains (chi-square analysis; **, *P* = 0.009). Our cohort consisted of a diverse array of *spa* types (17 different variants; see Table S1); however, only *spa* types t008 and t002 were frequent enough to merit statistical analysis. As with CC and *agr* types, neither *spa* type was significantly linked to CFZ responsiveness, while *spa* type t008 was significantly linked to OXA responsiveness (Table S1). Although our statistical analyses indicate that certain genotypic markers are more commonly associated with OXA-NaHCO_3_ responsiveness, our sample size is too small to draw any definitive predictive conclusions.

The majority of strains (59%) were either SCC*mec* type IV (59%) or SCC*mec* type II (38%) (Table S1), with no significant associations with NaHCO_3_ responsivity for CFZ or OXA.

It appears that the NaHCO_3_ responsiveness phenotype and sensitization of MRSA to two prototype standard-of-care β-lactams is relatively frequent, especially for CFZ. This phenotype does not appear to be related to the size of the resistant subpopulation or the intrinsic MIC of responsive strains. Although our data suggest that certain genotypic markers may be linked to OXA responsiveness, a larger cohort is needed to verify this observation.

Interestingly, a recent study by Quach et al. identified a subset of closely related MRSA strains, falling within the multilocus sequence type ST8 grouping (a lineage encompassed within CC8), that display unexpected high rates of lysis in the presence of β-lactams (21). This may point to one potential mechanism contributing to NaHCO_3_ responsiveness, i.e., NaHCO_3_-enhanced, β-lactam-mediated lysis. We are currently examining this phenotype among the 58 MRSA isolates described in the current study.

In summary, if the association of *in vitro* NaHCO_3_ responsiveness and β-lactam sensitization can be further validated experimentally *in vivo* using larger MRSA cohorts, a well-designed clinical trial to verify this relationship may well be justified.

## Supplementary Material

Supplemental file 1

Supplemental file 2
