# Supplementary material for: Scope and Predictive Genetic/Phenotypic Signatures of Bicarbonate (NaHCO3) Responsiveness and β-Lactam Sensitization in Methicillin-Resistant Staphylococcus aureus
Source: Antimicrob Agents Chemother. 2020 Apr 21;64(5):e02445-19. doi: 10.1128/AAC.02445-19 (PMC7179597; doi:10.1128/AAC.02445-19)
Supplement: Supplemental file 1 [file AAC.02445-19-s0001.pdf]

Figure S1

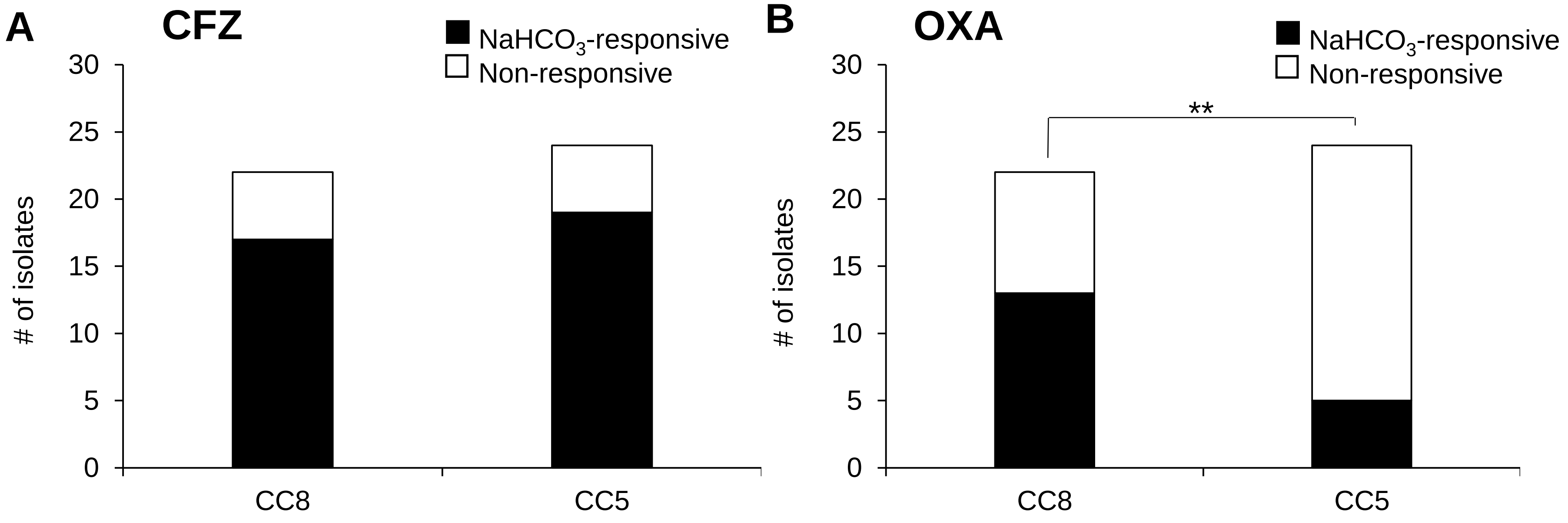

FIG S1. Relationship between CC types and responsiveness to CFZ and OXA. (**A**) Frequency of $NaHCO_3$-responsiveness to CFZ within the two most common CC types in cohort (CC8 and CC5). Neither CC8 nor CC5 was a better predictor of responsiveness to CFZ (Chi-squared analysis, P = 0.2). (**B**) Frequency of $NaHCO_3$-responsiveness to OXA within CC8 and CC5. CC8 was a better predictor of responsiveness to OXA than CC5 (Chi-squared analysis, **P = 0.008).
